# A series of vectors for inducible gene expression in multidrug-resistant *Acinetobacter baumannii*

**DOI:** 10.1128/aem.00474-24

**Published:** 2024-08-20

**Authors:** Valerie Intorcia, Rosa L. Sava, Grace P. Schroeder, Michael J. Gebhardt

**Affiliations:** 1 Department of Microbiology and Immunology, Carver College of Medicine, University of Iowa, Iowa City, Iowa, USA; Washington University in St. Louis, St. Louis, Missouri, USA

**Keywords:** apramycin, hygromycin, gene expression, plasmids, cloning

## Abstract

The continued emergence of antibiotic resistance among bacterial pathogens remains a significant challenge. Indeed, the enhanced antibiotic resistance profiles of contemporary pathogens often restrict the number of suitable molecular tools that are available. We have constructed a series of plasmids that confer resistance to two infrequently used antibiotics with variants of each plasmid backbone incorporating several regulatory control systems. The regulatory systems include both commonly used systems based on the *lac-* and arabinose-controlled promoters found in *Escherichia coli*, as well as less frequently used systems that respond to tetracycline/anhydrotetracycline and toluic acid. As a test case, we demonstrate the utility of these plasmids for regulated and tunable gene expression in a multidrug-resistant (MDR) isolate of *Acinetobacter baumannii*, strain AB5075-UW. The plasmids include derivatives of a freely replicating, broad-host-range plasmid allowing for inducible gene expression as well as a set of vectors for introducing genetic material at the highly conserved Tn7-attachment site. We also modified a set of CRISPR-interference plasmids for use in MDR organisms, thus allowing researchers to more readily interrogate essential genes in currently circulating clinical isolates. These tools will enhance molecular genetic analyses of bacterial pathogens in situations where existing plasmids cannot be used due to their antibiotic resistance determinants or lack of suitable regulatory control systems.

**IMPORTANCE:**

Clinical isolates of bacterial pathogens often harbor resistance to multiple antibiotics, with *Acinetobacter baumannii* being a prime example. The drug-resistance phenotypes associated with these pathogens represent a significant hurdle to researchers who wish to study modern isolates due to the limited availability of plasmid tools. Here, we present a series of freely replicating and Tn7-insertion vectors that rely on selectable markers to less frequently encountered antibiotics, apramycin, and hygromycin. We demonstrate the utility of these plasmid tools through a variety of experiments looking at a multidrug-resistant strain of *A. baumannii*, strain AB5075. Strain AB5075 is an established model strain for present-day *A. baumannii*, due in part to its genetic tractability and because it is a representative isolate of the globally disseminated multidrug-resistant clade of *A. baumannii*, global clone 1. In addition to the drug-selection markers facilitating use in strains resistant to more commonly used antibiotics, the vectors allow for controllable expression driven by several regulatory systems, including isopropyl β-D-1-thiogalactopyranoside (IPTG), arabinose, anhydrotetracycline, and toluic acid.

## INTRODUCTION

A key requirement for studying the molecular genetics of bacteria is the ability to controllably regulate gene expression. Indeed, controllable expression systems can facilitate complementation studies, the study of toxic proteins, or allow for researchers to conduct experiments aimed at understanding the consequences of depleting essential proteins. While numerous systems for controlling gene expression have been developed, these systems often have limited utility for interrogating isolates of multidrug-resistant (MDR) organisms due to the unavailability of suitable selection markers. The emerging opportunistic pathogen *Acinetobacter baumannii* highlights these difficulties. Many studies of *A. baumannii* biology have used the ATCC 17978 strain background, which was isolated in the 1950s. Meanwhile, in the past several years, there have been numerous reports highlighting that currently circulating clinical isolates of *A. baumannii* are more virulent and often harbor multiple antibiotic resistances (1–4). One such strain, AB5075-UW, has emerged as a model strain due to its genetic tractability (5–7), but, as a multidrug-resistant strain, many of the existing plasmid-based tools are unavailable due to incompatible origins of replication and/or limited selectable markers.

Here, we present a series of vectors that harbor different inducible expression systems to facilitate further exploration of molecular genetics in multidrug-resistant bacterial species. As a test case, we demonstrate the utility of these plasmids in a commonly used MDR isolate of *A. baumannii* isolate, strain AB5075-UW. We have developed both a series of freely replicating plasmids based on the broad-host range RSF1010 derivative, pMMB207 (8, 9) as well as a suite of vectors for integration of genetic material at the Tn7 attachment site on the chromosome (10). For each freely replicating or chromosomally integrating system, we have characterized multiple expression control systems, including systems responding to lactose/IPTG, arabinose, toluic acid, and tetracycline/anhydrotetracycline. To facilitate experimentation in bacteria harboring resistance to more commonly used antibiotics, the plasmids described herein confer resistance to two less commonly used antibiotics, apramycin (pMMB207-based plasmids) and hygromycin (Tn7-based plasmids), which expands the utility of this system for use in bacterial strains that are resistant to other commonly used selectable markers like kanamycin, gentamicin, and beta-lactams. We also modified the antibiotic resistance markers in a recently developed set of tools for CRISPR interference (CRISPRi; 11) to facilitate the study of essential genes in MDR bacterial isolates.

## RESULTS

### Replicating plasmids for use in multidrug-resistant organisms

**Fig 1:**
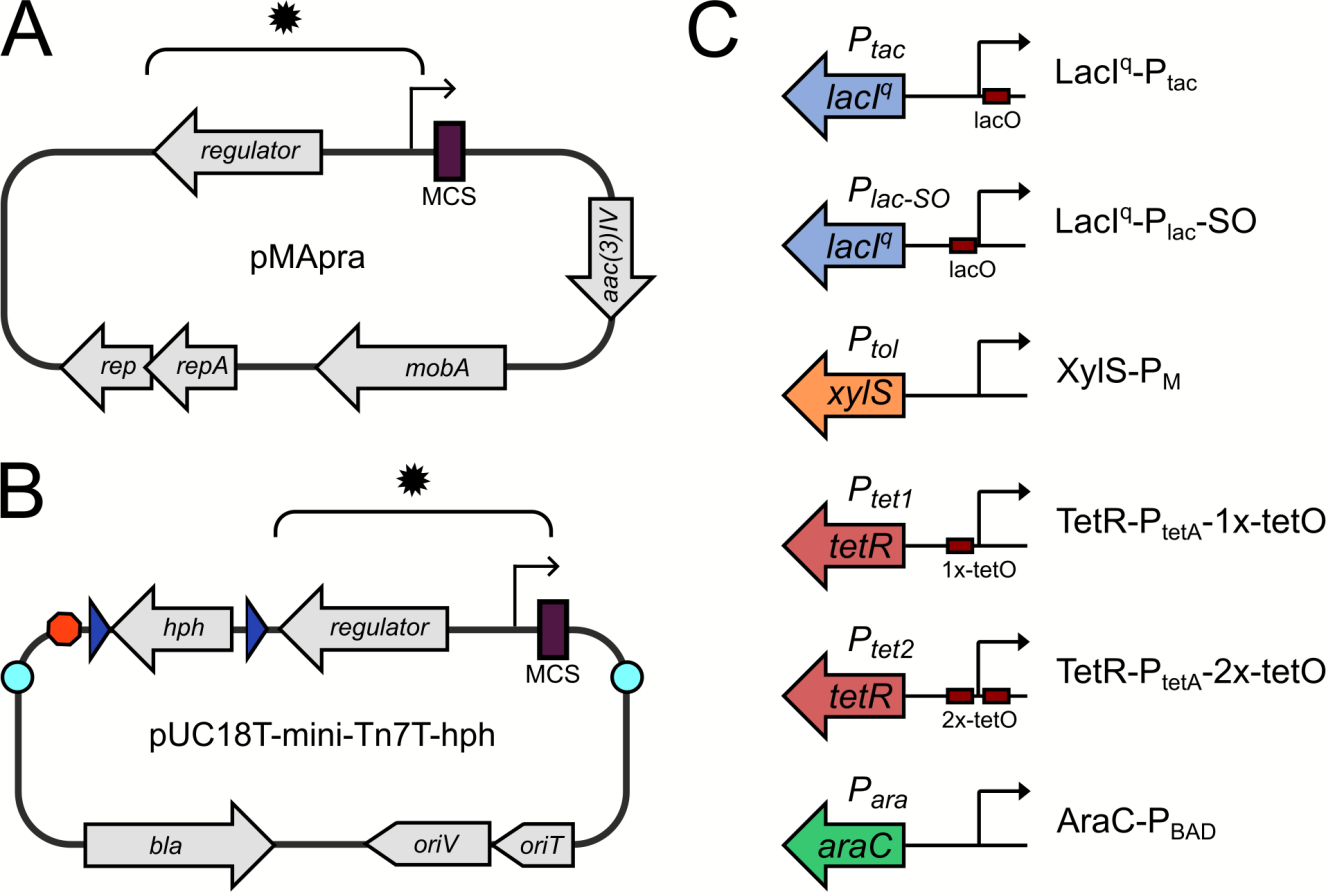
Plasmid architecture for replicating and Tn7-insertion plasmids. (**A**) Map of replicating plasmid backbone pMApra. (**B**) Map of Tn7-insertion site plasmids. (**C**) Schematic of regulatory systems. Asterisks in A and B indicate the location of the inserted regulatory systems. In B, blue arrows indicate Flp2 recombinase target sites, and a red octagon indicates transcription terminators (*E. coli rrnB* T1 terminator and lambda T0 terminator); light blue circles represent the left and right ends of the Tn7 cassette. MCS, multiple cloning site; *aac(3)IV*, apramycin resistance gene; *hph*, hygromycin resistance gene; *bla*, beta-lactamase resistance gene.

We previously created an incompatibility group Q plasmid that confers resistance to the aminoglycoside apramycin and contains the *lacI^q^*-Ptac regulatory system (12, 13). This plasmid, which we refer to herein as pMApra-Ptac, served as the vector backbone into which several additional regulatory control systems were added, including a modified *lac* promoter that contains a Lac repressor-binding site positioned between the −10 and −35 promoter elements (P*lac*-SO); a toluic acid-responsive system comprising the XylS-regulated Pm promoter together with the *xylS* gene from *Pseudomonas putida* (XylS-Pm, referred to as P*tol* hereafter); an arabinose inducible system comprising the AraC-regulated Pbad promoter together with the *araC* gene from *Escherichia coli* (AraC-Pbad, referred to as P*ara* hereafter); and two versions of a tetracycline-responsive system containing the TetR-repressed P*tetA* promoter together with the *tetR* gene from Tn10 and either one (TetR-PtetA-1x-tetO, referred to as P*tet1* hereafter) or two (TetR-PtetA-2x-tetO, referred to as P*tet2* hereafter) TetR repressor-binding sites, respectively (Fig. 1). The resulting plasmids (Table 1) each contain a multiple cloning site with several unique restriction sites to facilitate cloning (Fig. S1).

**TABLE 1:** pMApra plasmids

Name	Regulatory system	Inducer	Background^*a*^	Max expression^*b*^	Induction ratio^*c*^
pMApra-Ptac	LacI^q^/Ptac	IPTG	481 (±11)	32,970 (±2,883)	69×
pMApra-Plac-SO	LacI^q^/Plac derivative	IPTG	414 (±7)	11,823 (±1,837)	29×
pMApra-Ptol	XylS-Pm	*m-*toluic acid	360 (±10)	2,571 (±117)	7×
pMApra-Ptet1	TetR-PtetA with 1 TetO operator	aTc	499 (±24)	19,469 (±10,014)	39×
pMApra-Ptet2	TetR-PtetA with 2 TetO operators	aTc	353 (±13)	4,212 (±425)	12×
pMApra-Para	AraC-ParaBAD	Arabinose	415 (±7)	3,409 (±1,023)	8×

^*a*^ Values reflect mean relative fluorescence units (RFU) for mCherry at the 24-hour time point for triplicate cultures grown in the absence of an inducer. Values in parentheses indicate one SD of the mean.

^*b*^ Values reflect mean RFU for mCherry at the 24-hour time point for triplicate cultures grown in the presence of an inducer. Values in parentheses indicate one SD.

^*c*^ Induction ratio calculated by dividing the max expression value by background mCherry RFUs.

**Fig 2:**
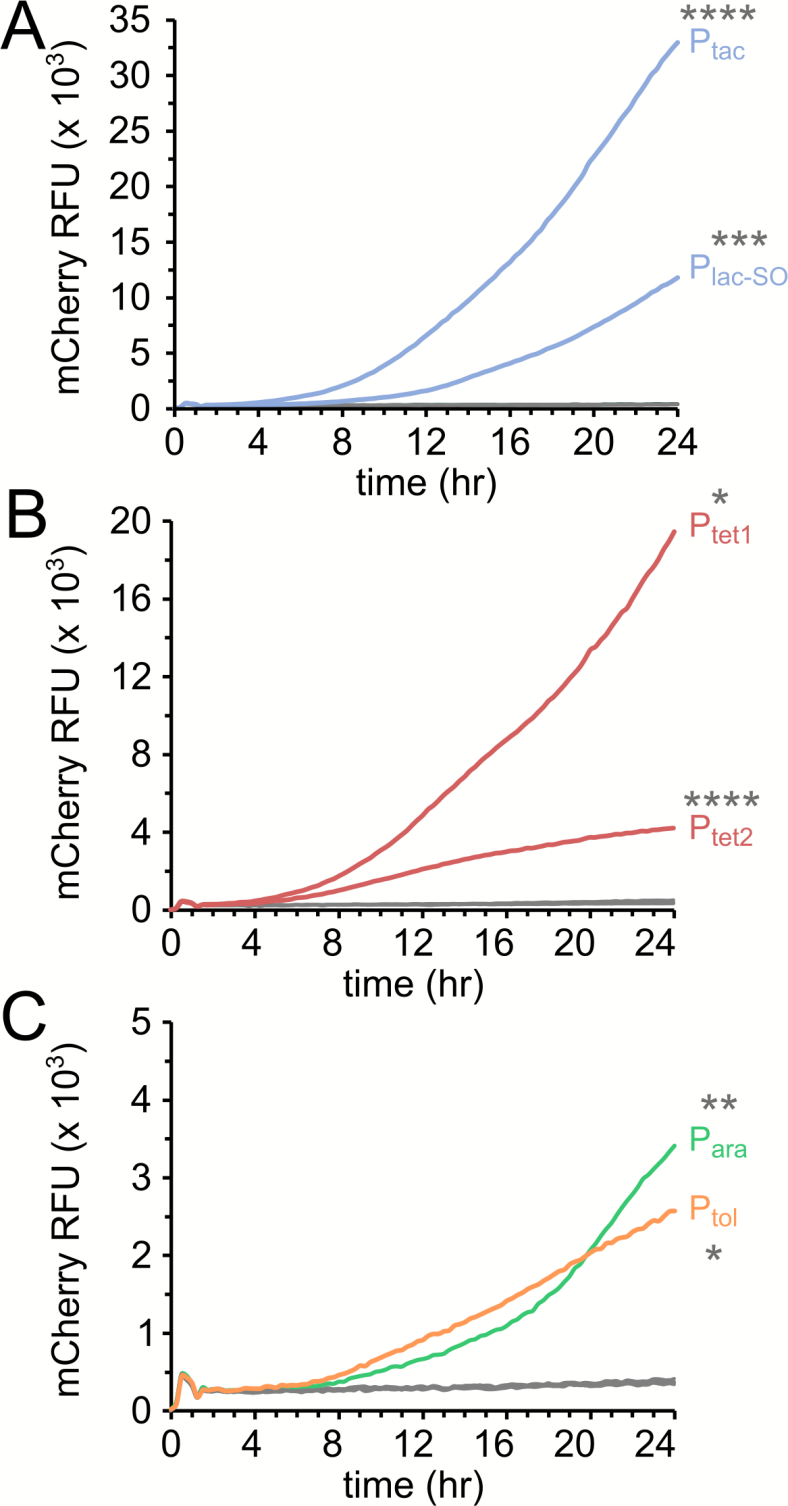
Activity of inducible systems expressed from replicating pMApra vectors. Graphs depict the relative mCherry fluorescence from growth curves in the presence and absence of inducers for IPTG-inducible vectors (**A**), tetracycline-inducible vectors (**B**), and arabinose- and toluic acid-inducible vectors (**C**). Gray lines depict the mCherry readings for each corresponding empty vector and growth in the absence of an inducer. Induction conditions were 2 mM IPTG for panel A, 50 ng/mL anhydrotetracycline for panel B, and 2% arabinose and 2 mM toluic acid for panel C. mCherry growth curves were performed with biological triplicate cultures and were repeated three times with independent transformations for each. Asterisks indicate the results of statistical comparisons between the induced and uninduced condition for each plasmid at the 24-hour time point. Statistics assessed via two-tailed *t* test; *, *P* < 0.05, **, *P* < 0.01; ***, *P* < 0.001; ****, *P* < 0.0001. Corresponding growth curves (optical density measurements) are shown in Fig. S2.

To demonstrate the functionality of these inducible systems, we introduced the coding sequence of a red fluorescent protein, mCherry, into each plasmid and assessed the response of each regulatory system to the corresponding inducer in *A. baumannii* strain AB5075-UW, a model multidrug-resistant clinical isolate (5, 14). As shown in Fig. 2 and Table 1, plasmid pMApra-P*tac*, which harbors the IPTG-inducible P*tac* promoter system, produced the highest expression of mCherry and an approximately 70-fold induction ratio between the induced and uninduced condition. The pMApra-P*tet1* plasmid generated the next highest amount of mCherry expression, yielding slightly more than half of that measured with pMApra-P*tac* vector and an induction ratio of 39-fold. The remaining vectors produced lower mCherry expression and induction ratios (Table 1; Fig. 2). The kinetics of induction were similar across all conditions tested, and there were no overt differences in the growth of the strains in the presence/absence of the inducers or vehicle controls, with the exception the pMApra-P*tol* plasmids, which showed a minor growth delay when toluic acid was added to the cultures (Fig. S2). Plasmid stability experiments indicated that the pMApra plasmid is stable when cultured in the absence of antibiotic selection; however, the stability decreased when cells were grown in the absence of selection concurrently with a high concentration of the inducer (Fig. S3).

### Plasmids for chromosomal insertion at the Tn7 attachment site

The Tn7-attachment site has been widely adopted as a stable site for the integration of genetic material in a diverse range of Gram-negative bacteria, including *A. baumannii* (10, 15, 16). We previously used the Tn7-based system to deliver a complementation construct for a transcription regulator called GigC (17). This previous Tn7-delivery plasmid (pMJG111) confers resistance to the aminoglycoside hygromycin and lacks a regulated promoter, thus requiring the inclusion of the gene’s native regulatory region. In the present study, we generated several additional versions of hygromycin-resistant Tn7-delivery plasmids that incorporate different regulatory systems, including those responding to IPTG, arabinose, toluic acid, and tetracycline/anhydrotetracycline. To do this, we first created hygromycin-resistant derivatives of plasmids pJM100 (pUC18T-mini-Tn7T-gm-araC-ParaBAD; referred to as Tn7-Para hereafter) and pJM101 (pUC18T-mini-Tn7T-gm-lacI^q^-Ptac; referred to as Tn7-Ptac hereafter) (18). We subsequently replaced the lacI^q^-Ptac cassette with either the toluic acid responsive (XylS-Pm; referred to as Tn7-Ptol hereafter) or tetracycline responsive (TetR-PtetA-1x-tetO; Tn7-Ptet1 hereafter and TetR-PtetA-2x-tetO; Tn7-Ptet2 hereafter) regulatory systems (Table 2; Fig. 1; Fig. S4).

**TABLE 2:** Tn7 plasmids

Name	Regulatory system	Inducer	Background^*a*^	Max expression^*b*^	Induction ratio^*c*^
pUC18T-mini-Tn7T-hph-Ptac	LacI^q^ / Ptac	IPTG	17.8 (±0.8)	5,096 (±228)	285×
pUC18T-mini-Tn7T-hph-Ptol	XylS-Pm	*m-*toluic acid	9.3 (±1.7)	2,141 (±391)	229×
pUC18T-mini-Tn7T-hph-Ptet1	TetR-PtetA with 1 TetO operator	aTc	13.9 (±1.5)	5,079 (±227)	365×
pUC18T-mini-Tn7T-hph-Ptet2	TetR-PtetA with 2 TetO operators	aTc	0.04 (±0.03)	66.5 (±6.6)	1,563×
pUC18T-mini-Tn7T-hph-Para	AraC-ParaBAD	Arabinose	33.4 (±2.9)	1,734 (±82.5)	52×

^*a*^ Values reflect mean LacZ activity (in Miller units) for three independent replicate experiments grown in the absence of an inducer. Values in parentheses indicate one SD of the mean.

^*b*^ Values reflect mean LacZ activity (in Miller units) for three independent replicate experiments grown in the presence of the highest inducer concentration tested. Values in parentheses indicate one SD.

^*c*^ Induction ratio calculated by dividing the max expression value by background Miller units.

**Fig 3:**
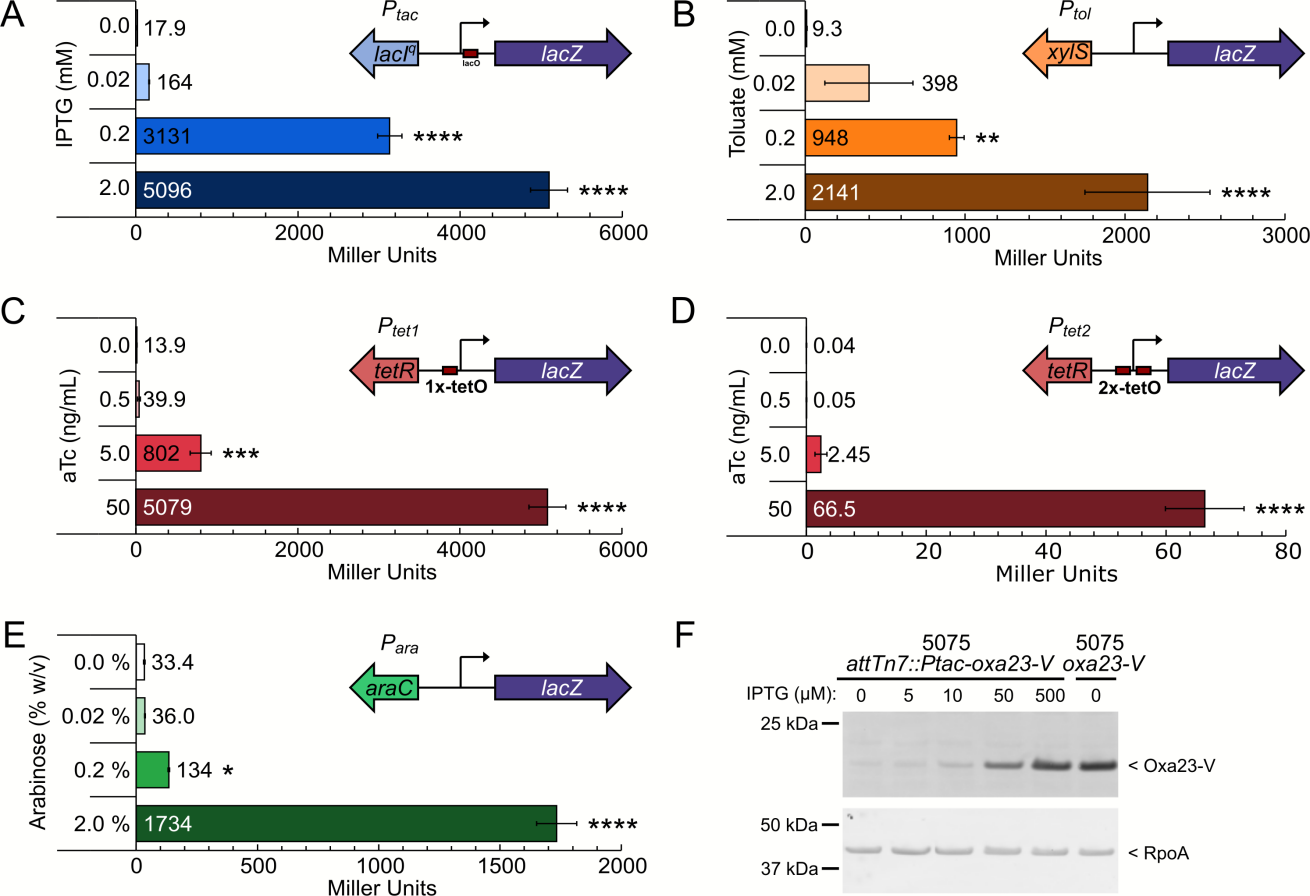
Activity of inducible systems expressed from the Tn7-site. (**A–E**) Beta-galactosidase assay results (data expressed as Miller units) for AB5075 harboring *lacZ* transcriptional fusions at the Tn7-attachment site for LacI^q^-Ptac (**A**), XylS-Pm (**B**), TetR-PtetA-1x-tetO (**C**), TetR-PtetA-2x-tetO (**D**), and AraC-ParaBAD (**E**). (**F**) Western blot analysis for lysates collected from cells of AB5075::attTn7-lacI^q^-Ptac*-oxa23-VSVG* or AB5075 *oxa23-VSVG*, which harbors a modified Oxa23 allele specifying a C-terminal vesicular stomatitis virus G (VSVG) epitope. Cells were collected from exponentially growing cultures in the presence of the indicated concentrations of IPTG. Insets in A–E depict a schematic of the *lacZ* transcriptional fusion construct tested. β-galactosidase assay data plotted as the average value (in Miller units) from three independent replicate experiments, and error bars represent one SD. Western blot samples were collected from biological duplicate cultures with results from a single replicate shown; the experiment was repeated independently three times with similar results. For A–E, statistics were assessed via one-way ANOVA with Dunnett’s post-hoc test to correct for multiple comparisons. Asterisks indicate significant differences relative to the activity measured in the absence of inducer; *, *P* ≤ 0.05; **, *P* < 0.01; ***, *P* < 0.001; ****, *P* < 0.0001.

To test these Tn7 delivery plasmids with their respective inducing substances, we generated transcriptional fusion reporters, where the *lacZ* gene was inserted downstream of the regulated promoter. Each of the Tn7 lacZ-reporter plasmids was introduced into wild-type AB5075 cells by four-parental mating as described previously (12, 19) and in Materials and Methods. The resulting cells were grown in lysogeny broth (LB) to the exponential phase (OD_600_ ≈ 0.4–0.6) in the presence of increasing concentrations of each system’s corresponding inducing molecule, and beta-galactosidase assays were performed to assess the response of each regulatory system. As shown in Fig. 3 and Table 2, for each *lacZ* fusion, growth in the presence of the corresponding inducing substance led to dose-dependent increases in *lacZ* expression, as assessed by standard β-galactosidase/Miller assays. The Tn7-Ptac and Tn7-Ptet1 *lacZ* reporters, which are induced by IPTG and anhydrotetracycline (aTc), respectively, each produced a maximum activity of ≈5,000 Miller units, with the Tn7-Ptac reporter showing a more linear dose-response to increasing concentration of IPTG but had an overall lower induction ratio when comparing activity in the presence and absence of inducer with a 285-fold induction for Tn7-Ptac compared to approximately 365-fold induction for Tn7-Ptet1. Indeed, the Tn7-Ptet1 reporter had slightly lower basal activity and a more graded response in the presence of lower concentrations of inducer. The toluic acid responsive *lacZ* reporter, driven by Tn7-Ptol, responded in a dose-dependent fashion to its inducer with a low basal activity and a lower maximal induction of 2,300 Miller units giving an induction ratio of 230-fold. The arabinose responsive *lacZ* reporter (Tn7-Para) had the highest basal expression in the absence of inducer (33 Miller units) and lowest maximal induction of approximately 1,700 Miller units with a 52-fold increase in reporter activity in the presence of maximal inducer concentration. The Tn7-Ptet2 promoter showed the lowest activity amongst the tested reporters and cells produced only ≈60 Miller units at the maximal inducer concentration tested. The Tn7-Ptet2-driven *lacZ* reporter also had the lowest basal activity, with minimal activity detected (less than 5 Miller units) when no or low concentrations (less than 0.5 ng/mL) of aTc were added. The very low basal activity, which likely accounts for the very high observed induction ratio (≈1,500-fold induction), indicates that single-copy integration of the Tn7-Ptet2 regulatory system is very tightly repressed in the absence of its inducer.

We also created a Tn7 plasmid to allow for controlled expression of an epitope-tagged protein of interest. In the Tn7-Ptac regulated plasmid, we introduced an in-frame copy of the vesicular stomatitis virus G (VSVG) protein epitope. The VSVG epitope is positioned immediately 3′ to a NotI restriction site to allow for the introduction of a 3-alanine linker between the open reading frame of interest with that of the VSVG epitope. Into the resulting plasmid, we cloned the coding sequence for Oxa23, a key determinant of carbapenem resistance in the AB5075 strain (20). We introduced the Tn7-Ptac-Oxa23-VSVG cassette into wild-type AB5075 at the Tn7 attachment site and assessed the production of Oxa23 of the resulting cells in response to increased concentrations of IPTG by western blot using anti-VSVG antibodies. As can be seen in Fig. 3F and consistent with the β-galactosidase assays discussed above, cells grown in the presence of increasing concentrations of IPTG generated increasing amounts of VSVG-tagged Oxa23 as determined by western blot.

### Plasmids for CRISPR-interference

A CRISPRi system was recently adapted for use in *A. baumannii* (11). The system utilizes a nuclease-defective Cas9 enzyme (dCas9) under the control of the Ptet2 (i.e., TetR-PtetA-2x-tetO) regulatory system via integration at the Tn7-attachment site. In addition, CRISPRi requires a single-guide RNA (sgRNA) for targeting the dCas9 protein to the gene of interest. To adapt this CRISPRi system for use in MDR *A. baumannii* isolates, we modified the Tn7/dCas9 delivery plasmid to confer resistance to hygromycin and modified the sgRNA plasmid pYDE07, which expresses the sgRNA from the constitutive J23119 promoter (21) to confer resistance to apramycin (Fig. S5).

**Fig 4:**
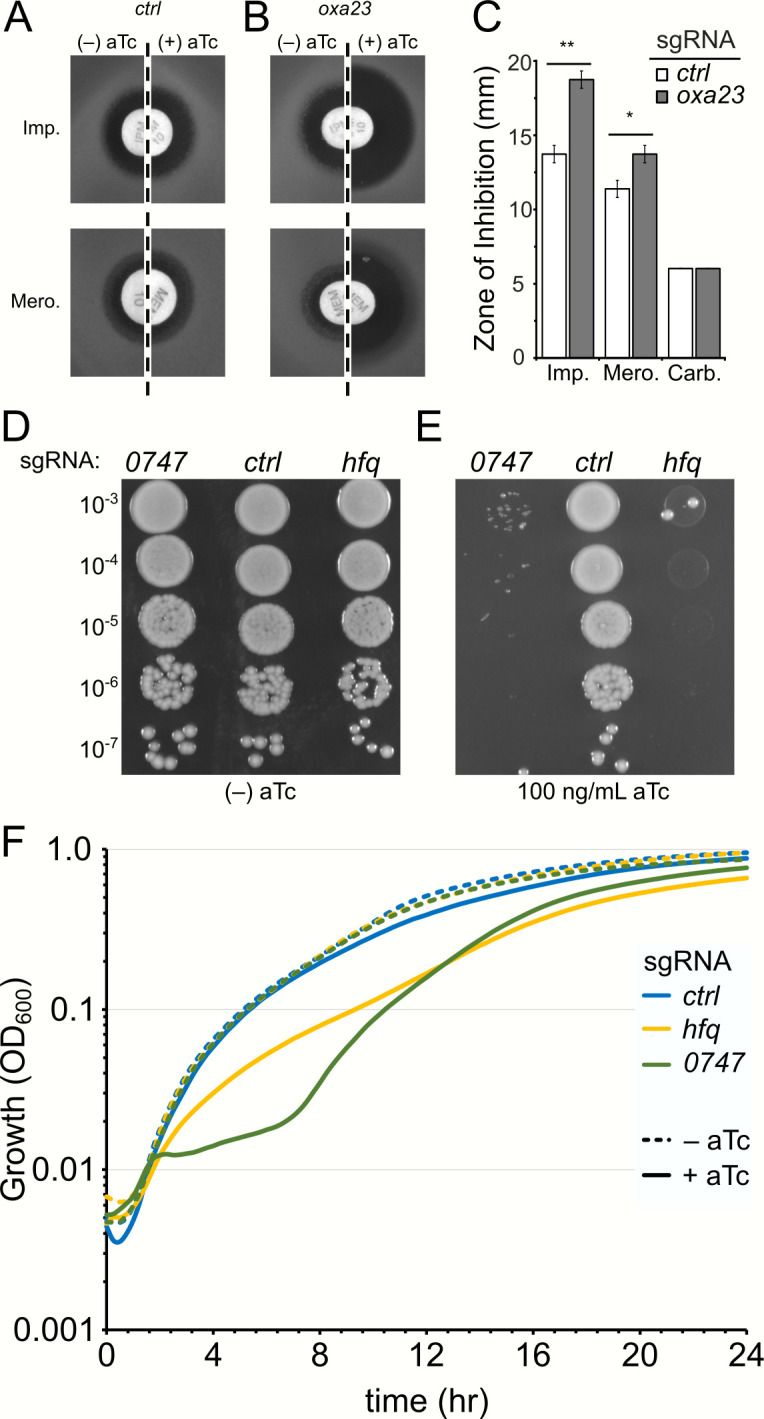
CRISPRi in AB5075. sgRNA expression plasmids (pWH1266-Apra-sgRNA) were transformed into an AB5075 derivative containing a tetracycline-regulated *dCas9* cassette (TetR-PtetA-2x-tetO) at the Tn7 attachment site. Overnight cultures were plated onto LB agar plates containing apramycin with or without the addition of 100 ng/mL of anhydrotetracycline (aTc) for *dCas9-*expressing cells expressing either a control (**A**) or *oxa23-*targeting sgRNA (**B**). Filter discs containing antibiotics were added, and the plates were incubated for ≈18 hours at 37°C. (**C**) Quantification of zones of inhibition from A and B. (**D and E**) *CRISPRi* targeting essential genes inhibits growth. Colonies containing the indicated sgRNA plasmids were suspended in PBS, serially diluted, and spotted on to LB agar plates containing apramycin with (panel D) or without (panel E) the addition of 100 ng/mL of aTc to induce *dCas9* expression. Plates were dried at room temperature and incubated at 37°C overnight (≈18 hours). (**F**) Overnight cultures were diluted into fresh LB media containing apramycin and either DMSO (–aTc) or 50 ng/mL aTc (+aTc) and grown in a plate reader at 37°C. Growth was recorded as OD_600_ every 15 min for 24 hours. Each sgRNA plasmid was tested with triplicate wells in biological duplicate. A representative experimental run is shown for one replicate, depicting the mean OD_600_ reading for the triplicate wells. Error bars are omitted for clarity. All experiments were repeated at least three times on separate days with fresh transformation of sgRNA-delivery plasmid with representative data shown. Asterisks in panel C indicate statistical significance as assessed by two-tailed *t* test comparing the zone of inhibition for the control sgRNA and the sgRNA targeting *oxa23*, **, *P*-value ≤ 0.001, *, *P*-value < 0.01. Imp., imipenem; Mero., meropenem; Carb., carbenicillin.

We introduced the dCas9 expression cassette into wild-type AB5075 at the Tn7-attachment site and, following induction with aTc, detected synthesis of dCas9 by western blot analysis (Fig. S5). We subsequently designed CRISPRi sgRNAs to target three genes in the AB5075 genome including *oxa23*, a key contributor of carbapenem resistance in AB5075, and two putative essential genes, *hfq* and *ABUW_*0747, which encode for a key RNA chaperone and a Cro/CI family transcription factor, respectively (2, 11). We induced dCas9 expression in cells harboring either a control sgRNA (targeting the coding sequence of mCherry) or an sgRNA targeting the non-template strand of *oxa23* and assessed resistance to two carbapenem antibiotics, imipenem and meropenem, as well as carbenicillin, a penicillin-class antibiotic. CRISPRi-mediated depletion of Oxa23 led to a significant increase in sensitivity against both carbapenems (Fig. 4). We also assessed the ability of the CRISPRi system to target essential genes. We assayed the CRISPRi system both in liquid growth media and on solidified agar plates. The expression of dCas9 in cells harboring guide RNAs targeting either *hfq* or *ABUW_0747* led to significantly impaired growth when spotted on plates containing anhydrotetracycline (Fig. 4D and E). Additionally, delayed and altered growth kinetics were observed in liquid culture only in the presence of the inducer (Fig. 4F). Suppressors frequently arose following CRISPRi induction, particularly in liquid culture (Fig. 4F); re-sequencing the guide RNA plasmids for these cultures indicated that several had acquired mutations in the sgRNA sequence or promoter region on the plasmid.

## DISCUSSION

Here, we describe a suite of plasmids for controlled gene expression in *A. baumannii* using both a multi-copy approach (i.e., pMApra vectors) and a single-copy chromosomal insertion approach (i.e., integration at the Tn7 attachment site). The regulatory control systems allow for the use of the commonly used inducers, IPTG and arabinose, as well as anhydrotetracycline, and the less commonly used inducer toluic acid. The lacI^q^-Ptac regulatory system produced the highest expression levels in both the replicating plasmid (Fig. 2) and when integrated at the Tn7 site (Fig. 3). The toluic acid system produced very low basal expression in the uninduced condition (Fig. 2 and 4), although AB5075 cells harboring the pMApra-XylS-Pm based plasmids also showed a modest growth defect in the presence of toluic acid (Fig. S2), suggesting that toluic acid may impart some toxicity to *A. baumannii*. For the arabinose system, maximal expression was achieved in growth media containing 2% (wt/vol) arabinose, a concentration which may cause unintended physiological consequences, although we did not explicitly observe altered growth kinetics under the experimental conditions tested herein (Fig. S2). The two configurations of the tetracycline-responsive plasmids allow for broad ranges of expression, with strong induction with the Ptet1 promoter and very tight regulatory control when using the Ptet2 promoters. The tight repression achieved by the Tn7-based Ptet2 construct is particularly well-suited for interrogating essential genes using CRISPRi (Fig. 4), which we demonstrate by specifically repressing transcription of two candidate essential genes, *hfq* and *ABUW_0747* (Fig. 4). In addition, the Tn7-based Ptet2 promoter would be ideal for numerous experimental approaches which require tight transcriptional control, such as experiments to demonstrate synthetic lethality or inducer-wash out experiments to deplete a target protein as cells divide. This system could likewise be readily adapted to study the consequence of expressing acutely toxic gene products, such as dominant negative protein alleles or the toxin components of toxin/anti-toxin systems. One cautionary note, however, for the CRISPRi system is that suppressing mutations in guide RNA plasmids arose, particularly with cells cultured in liquid media over a 24-hour period (Fig. 4F). This observation suggests that experimental results from such long-term induction periods with this CRISPRi system should be interpreted with caution.

Several labs have developed tools, using a variety of expression/induction systems, for use in MDR bacterial pathogens, including *A. baumannii*. We and others have taken advantage of an *Acinetobacter* specific replicon, pWH1266, to create *E. coli-Acinetobacter* shuttle vectors based on the origin of replication from a native *Acinetobacter* plasmid and *E. coli-*compatible plasmid origins (i.e., pBR322 or pColE1) (22, 23). The sgRNA plasmid described herein is a derivative of pWH1266, and one limitation we have observed with this plasmid is the potential for toxicity in *E. coli* due to the high copy number of the pBR322 backbone and high expression from the strong promoter used to drive sgRNA expression. Wu and colleagues used a constitutive lac promoter in their investigation of the Oxa23 carbapenemase (20). One potential limitation to this approach is that constitutive expression may prove problematic when studying genes or gene products that are toxic when highly expressed (24). Another approach to solve the limited availability of antibiotic markers has been to construct strains harboring large deletions to remove known antibiotic resistance loci from otherwise MDR clinical isolates, as has been done with *A. baumannii* strain AB5075 (2). This approach also has limitations, as there are often regulatory elements embedded within these resistance cassettes, such as small non-coding RNA elements or transcription regulators (7, 25), that may exert global effects on gene expression.

We report here the construction of a suite of plasmids allowing for regulated gene expression either as a freely replicating plasmid or via the stable integration of genetic material at the Tn7-attachment site. The broad-host range of the pMApra backbone (RSF1010/plasmid incompatibility group Q) and the utility of the Tn7 system across diverse bacterial species should also be beneficial for investigators who are interested in other organisms. Indeed, we and others have utilized pMMB-based replicon plasmids in a diverse collection of bacterial species, including, but not limited to *Acinetobacter*, *Caulobacter*, *Francisella*, *Klebsiella*, *Legionella*, *Mycobacterium*, *Pseudomonas*, *Salmonella*, and *Vibrio* (26–28 and references therein). Likewise, the Tn7-attachment site has been widely exploited across Gram-negative bacteria to construct stable, chromosomal insertion of genetic material (10, 15, 16, 29, 30). Furthermore, the two sets of vectors described herein are compatible with each other, a feature that affords experimental flexibility by allowing for the simultaneous control of multiple replicons. These tools can be used to facilitate further investigation into multidrug-resistant bacteria and provide a resource to allow for more complex experimental approaches to understand the molecular genetics and physiology of contemporary bacterial pathogens.

## MATERIALS AND METHODS

### Bacterial strains and growth conditions

Bacterial strains are listed in Table S1. *A. baumannii* strain AB5075-UW was routinely grown in LB (10 g/L NaCl, 10 g/L tryptone, and 5 g/L yeast extract) at 37°C with shaking at 200 rpm, or on LB agar (15 g/L bacto agar) plates. As needed, antibiotics were added to growth media at the following concentrations: apramycin, 50 µg/mL (solid) and 25 µg/mL (liquid); hygromycin, 250 µg/mL; chloramphenicol, 5 µg/mL; tetracycline, 10 µg/mL; kanamycin, 50 µg/mL (solid) and 25 µg/mL (liquid); carbenicillin 100 µg/mL (solid) and 50 µg/mL (liquid). Anhydrotetracycline (Fisher Scientific cat. no. 10009542) was prepared at a stock concentration of 1 mg/mL in dimethyl sulfoxide (DMSO) and diluted into LB media to the final working concentrations as indicated; an equivalent dilution of DMSO was performed to serve as the vehicle control. *m*-Toluic acid (Sigma Aldrich cat. no. T36609) was prepared in dimethylformamide (DMF); all experiments with toluic acid included an equivalent amount of DMF as the vehicle control.

### Plasmid construction

Plasmids used and created in this study are listed in Table S2. Oligonucleotide primers are listed in Table S3. Molecular cloning of plasmids was performed in *E. coli* strain DH5α. Plasmid backbones were digested with restriction enzymes as recommended by the manufacturer (NEB), dephosphorylated with QuickCIP (NEB cat. no. M0525S), and purified following agarose gel electrophoresis. PCR products were amplified using KOD Extreme Host Start DNA Polymerase (Sigma Aldrich cat. no. 71975) in 50 µL reactions according to the manufacturer’s recommendations. The oligonucleotide primers used for cloning purposes contained 20–25 nt of homologous bases at the 5′ ends to facilitate cloning by isothermal assembly (ITA) (31). ITA reactions were performed at 50°C for 20 minutes prior to the heat-shock transformation of the assembly reaction into chemically competent DH5α. All plasmid inserts were verified by sequencing at the Iowa State University DNA Facility. A detailed description of plasmid construction can be found in the supplemental materials. The empty vectors described herein have been deposited with and are available from the Addgene vector repository.

### Preparation and transformation of electrocompetent *A. baumannii*

To prepare electrocompetent *A. baumannii* cells, 1.5 mL of overnight cultures were centrifuged in sterile microcentrifuge tubes and washed three times with 1 mL of 0.3 M sucrose. The final washed cell pellet was suspended in 400 µL 0.3 M sucrose, and 70 µL of the cell suspension was used for electroporation of 50–100 ng of plasmid DNA (pMApra and pWH1266-Apra derivatives). Electroporation was performed using a BIORAD Micropulser on setting EC2. Immediately following electroporation, cells were allowed to recover in 900 µL LB at 37°C for 60 minutes prior to plating on the appropriate selective media.

### Introduction of Tn7 plasmids to *A. baumannii*

Tn7-delivery plasmids were introduced into *A. baumannii* AB5075 by four-parental mating as described previously (12). Briefly, 0.1 mL of each overnight culture, including *A. baumannii* AB5075 (recipient), *E. coli* strain LW264 harboring the Tn7-delivery plasmid (donor), *E. coli* strain TOP10 pRK2013 (helper), and *E. coli* DH5α(λpir) pTNS3 (Tn7-transposon helper), was mixed and centrifuged at 7,000 × *g* for 2 minutes. The supernatant was removed, and the cell pellet was washed with 0.5 mL of fresh LB medium and pelleted again. The resulting cell pellet was suspended in 30 µL of LB and spotted on an LB agar plate. The liquid was allowed to dry at room temperature after which the plates were incubated at 37°C for approximately 8 hours. Growth patches were subsequently collected in 1 mL of sterile phosphate buffered saline (PBS) and plated onto LB plates with hygromycin and chloramphenicol to select for transconjugants. Proper integration of the Tn7 cassette was verified by colony PCR.

### Construction of *A. baumannii* mutant strains

*A. baumannii* mutant strains were constructed using the allele exchange vector pMJG42, which confers resistance to tetracycline and sensitivity to sucrose for counterselection (12). The desired mutation constructs were generated through PCR using the oligonucleotide primers listed in Table S3, which were then cloned into pMJG42. The resulting pMJG42 derivatives were mated into recipient *A. baumannii* cells using *E. coli* strain LW264 as the donor. Transconjugants were selected on LB plates containing tetracycline (10 ng/µL) and chloramphenicol (5 ng/µL) and were subsequently grown on yeast-tryptone plates (5 g/L yeast extract, 10 g/L tryptone, and 15 g/L bacto agar) containing 5% (wt/vol) sucrose to select for clones that had resolved the pMJG42 plasmid backbone. Sucrose-resistant colonies were screened by colony PCR and/or DNA sequencing to identify clones harboring the desired mutation.

### Beta-galactosidase assays

Beta-galactosidase assays were conducted essentially as previously described (32). Overnight cultures of the indicated reporter strain were diluted 1:500 in fresh growth media containing the indicated vehicle control or inducing substance and incubated with shaking at 37°C until mid-exponential phase (OD_600_ of approximately 0.4–0.6; approximately 5 × 10^8^ CFU/mL). Bacterial cells were permeabilized with chloroform and 0.1% SDS and assayed for β-galactosidase activity using 2-nitro-phenyl-β-D-galactopyranoside [Research Products International (RPI) cat. no N81000]. β-galactosidase assays were repeated independently at least three times with biological triplicate cultures; the results are plotted as the average value from three replicate experiments, with error bars representing one SD. Statistical analyses were conducted by one-way analysis of variance (ANOVA) with Dunnett’s post-hoc test to correct for multiple comparisons using GraphPad Prism version 10.2.

### Western blotting

For the Oxa23-VSVG western blot, overnight cultures of AB5075::Tn7-Ptac-Oxa23-V or an AB5075 *oxa23-VSVG* derivative, which encodes a modified allele of *oxa23* specifying a C-terminal VSV-G epitope tag from the chromosomal *oxa23* locus, were back diluted into LB media containing the indicated concentrations of IPTG at a starting density of 0.01 OD_600_ units. The cultures were grown with shaking at 37°C to exponential phase (≈3.25 hours; OD_600_ of approximately 0.4–0.6, corresponding to ≈5 × 10^8^ CFU/ml), at which point 500 µL of cells were pelleted. Cell pellets were dissolved in 1× LDS Sample Buffer (Thermo Fisher cat. no. NP0007) and resolved through a 12% Bis-Tris NuPAGE gel (Thermo Fisher cat. no. NP0321) in 1× MOPS running buffer (Thermo Fisher cat. no. NP0001). Proteins were transferred to Immobilon-PSQ polyvinylidene fluoride membrane (EMD Millipore cat. no. ISEQ08100) using an XCell-II Blot Module (Thermo Fisher). Membranes were fixed with methanol and blocked for at least 1 hour in a 1:5 dilution of blocking buffer (LI-COR cat. no. 072–70001) in PBS before being probed for 1 hour with rabbit anti-VSVG (1:3,333 dilution in diluted blocking buffer; Sigma Aldrich cat. no. V4888) and mouse anti-RpoA antibodies (1:10,000 dilution in diluted blocking buffer, BioLegend cat. no. WP003). The membranes were washed four times with a wash buffer comprised of 1× PBS + 0.05% Tween (Sigma Aldrich cat. no. P1379), blocked again for 15 minutes, and probed with donkey anti-rabbit IgG secondary antibody conjugated with the near-infrared (IR) dye 800CW (1:15,000 dilution in wash buffer; LI-COR cat. no. 925–32213) and goat anti-mouse IgG secondary antibody conjugated with the near-IR dye 680RD (1:30,000 dilution in wash buffer; LI-COR cat. no. 925–68070) for 1 hour. The membranes were washed four times with wash buffer and two times with 1× PBS, dehydrated with methanol, and imaged using an Azure Sapphire Imaging System (Azure Biosciences). Western blot experiments were independently repeated three times, and the results from a single representative experiment are shown.

### CRISPRi plate assay

Single colonies of AB5075-Tn7::*dCas9* strain harboring the indicated pWH1266-Apra-sgRNA expression plasmid were suspended in 100 µL of sterile PBS and subjected to serial 10-fold dilutions. Subsequently, 10 µL aliquots of the indicated dilutions were spotted onto LB plates with apramycin with or without 100 ng/mL aTc, dried at room temperature, and incubated at 37°C overnight (≈16–18 hours). Three biological replicates for each sgRNA were included in each experimental replicate with results of a single representative replicate shown. The experiment was repeated at least three times with independent electroporations.

### Disc diffusion assays

Triplicate overnight cultures (0.05 mL) of the AB5075-Tn7::*dCas9* strain harboring pWH1266-Apra-sgRNA derivatives expressing a control sgRNA (mCherry) or an *oxa23-*targeting sgRNA were mixed with 4 mL molten LB-top agar (LB media with 0.75 g/L bacto agar) and poured onto an LB plate containing apramycin (50 µg/mL) with or without 100 ng/mL aTc. The agar overlay was allowed to solidify, and filter discs containing 10 µg meropenem (BD cat. no. 231703), 10 µg imipenem (BD cat. no. 231644), or carbenicillin (5 µL of 100 mg/mL stock carbenicillin solution, RPI cat. no. C46000) were placed on top of the agar surface. The plates were incubated at 37°C for approximately 16–18 hours, and the diameter of the zone of growth inhibition surrounding each disc was measured. Data are plotted as the mean value from biological triplicate cultures with error bars representing one SD. Statistical significance was assessed via a two-tailed *t* test. Disc diffusion assays were conducted with biological triplicate cultures and were repeated three times; the results from a single representative experiment are shown.

### Growth curves

Bacterial growth curves were conducted in a 96-well dish with triplicate wells for each bacterial strain/plasmid condition. Briefly, overnight cultures were refreshed at a final dilution of 1:200 in 200 µL of growth media (LB) with the appropriate antibiotics and vehicle control/inducers. The plates were incubated in a Tecan M200 microplate reader at 37°C with growth and/or mCherry readings recorded every 15 minutes for 24 hours. The plates were shaken for 5 seconds prior to each read. Growth was recorded as the optical density at 600 nm (OD_600_). Fluorescent intensity of mCherry was measured with excitation/emission wavelengths of 587 nm and 610 nm, respectively. Data are plotted as the mean OD_600_ value for triplicate wells, and the growth curve experiments were repeated at least three times; the results of a representative experiment are shown. Statistical analyses to compare induced vs non-induced conditions for each plasmid system were conducted via two-tailed *t* test at the 24-hour time point.

## Data Availability

The plasmids described herein have been deposited in the Addgene plasmid repository.

## SUPPLEMENTAL MATERIAL

Supplemental material aem.00474-24-s0001.docx Supplemental methods, Figures S1 to S5, and Tables S1 to S5.

ASM does not own the copyrights to Supplemental Material that may be linked to, or accessed through, an article. The authors have granted ASM a non-exclusive, world-wide license to publish the Supplemental Material files. Please contact the corresponding author directly for reuse.
