# Supplementary material for: A series of vectors for inducible gene expression in multidrug-resistant Acinetobacter baumannii
Source: Appl Environ Microbiol. 2024 Aug 20;90(9):e00474-24. doi: 10.1128/aem.00474-24 (PMC11409637; doi:10.1128/aem.00474-24)
Supplement: Supplemental material — Supplemental methods, Figures S1 to S5, and Tables S1 to S5. [file aem.00474-24-s0001.docx]

A series of vectors for inducible gene expression in multidrug-resistant *Acinetobacter baumannii*

**Supplemental Methods**

*Plasmid Construction*

Unless noted otherwise, plasmid construction was routinely performed using isothermal assembly (1). Briefly, PCR products were generated using KOD Extreme polymerase (Sigma Aldrich cat. no. 71975-M) according to the manufacturer’s specifications and purified following agarose gel electrophoresis. Oligonucleotide primers (Table S3) used for ITA-based cloning were designed to incorporate 20-25 nt homologous DNA to the target plasmid insertion site. In the instances where synthetic DNA was used as the plasmid insert, the DNA was ordered as a gBlock from IDT (Coralville, IA), hydrated according to the manufacturer’s recommendations and combined with the digested plasmid backbone via ITA. Plasmid inserts were sequenced by the Iowa State University DNA facility (sanger sequencing) or Plasmidsaurus (whole – plasmid sequencing) to confirm the plasmids were assembled correctly and free of mutations.

*Empty vectors for multicopy plasmids.*

The apramycin resistant, broad host-range plasmid pMJG120, referred to herein as pMApra-Ptac, served as the parent vector backbone for construction of the araC-ParaBAD plasmid, pMApra-Para (Gebhardt lab plasmid pMJG464); the remaining regulatory systems were subcloned into this vector. To create pMApra-Para, pMApra-Ptac was digested with AseI and BsaI and combined with a PCR product containing the AraC-ParaBAD cassette from plasmid pKH6, which was amplified with primers MJG2442 and MJG2443. Primer MJG2443 also incorporates a tR’ terminator sequence. The pMApra-Plac-SO (Gebhardt lab plasmid pMJG584) contains lacI^q^ and a derivative of the *lac* promoter with a lac repressor binding site positioned between the -35 and -10 elements of the promoter (promoter sequence: TTGA*AATTGTGAGCGGATAACAATT*ATAAT; -35 and -10 elements underlined; lac operator in italic font) was constructed by first digesting plasmid pMApra-Para with AseI and PstI and inserting into this two PCR products: the first contains the *lacI^q^* sequence from pMApra-Ptac (amplified with primers MJG2874 and MJG2875) and the second contains the modified promoter sequence (amplified with primers MJG2878 and MJG2878; using gBlock 'two_operator_v1’ as the template). The toluic-acid responsive plasmid pMApra-Ptol (Gebhardt lab plasmid pMJG588) was created by inserting a PCR product generated with primers MJG2987 and MJG2989 (using plasmid pTOL as PCR template) into plasmid pMApra-Para that had been digested with AseI and XbaI. Plasmid pMApra-Ptet1 (Gebhardt lab plasmid pMJG598) was created by inserting a PCR product generated with primers MJG2917 and MJG2918 (plasmid pYDE09 was used for template) into plasmid pMApra-Para that had been previously digested with AseI and XbaI. Plasmid pMApra-Ptet2 (Gebhardt lab plasmid pMJG599) was created by inserting a PCR product generated with primers MJG2917 and MJG2919 (plasmid pYDE09 was used for template) into plasmid pMApra-Para that had been previously digested with AseI and XbaI.

*mCherry Expression Vectors*

Plasmid pMApra-Ptac-mCherry (Gebhardt lab plasmid pMJG116) was created by subcloning an mCherry fragment, including a strong ribosome binding site from plasmid pXDC18.mCherry. The mCherry fragment was released from pXDC18.mCherry by digestion with EcoRI and XbaI and was ligated into plasmid pMApra-Ptac digested with the same enzymes. Plasmid pMApra-Plac-SO-mCherry (Gebhardt lab plasmid pMJG596) was created by inserting the mCherry expression cassette (amplified with primers MJG2848 and MJG2849, using pMApra-Ptac-mCherry as template) into plasmid pMApra-Plac-SO digested with EcoRI and HindIII. Plasmid pMApra-Para-mCherry (Gebhardt lab plasmid pMJG484) was created by inserting the mCherry expression cassette (amplified with primers MJG2570 and MJG2571, using pMApra-Ptac-mCherry as template) into plasmid pMApra-Para that had been digested with XbaI and HindIII. Plasmid pMApra-Ptol-mCherry (Gebhardt lab plasmid pMJG602) was created by inserting the mCherry expression cassette (amplified with primers MJG2904 and MJG2849, using pMApra-Ptac-mCherry as template) into the XbaI/HindIII site of plasmid pMApra-Ptol. Plasmid pMApra-Ptet1-mCherry (Gebhardt lab plasmid pMJG603) was created by inserting the mCherry expression cassette (amplified with primers MJG2923 and MJG2849, using pMApra-Ptac-mCherry as template) into plasmid pMApra-Ptet1 that had been digested with DraI and HindIII. Plasmid pMApra- Ptet2-mCherry (Gebhardt lab plasmid pMJG604) was created by inserting the mCherry expression cassette (amplified with primers MJG2924 and MJG2849, using pMApra-Ptac-mCherry as template) into plasmid pMApra-Ptet2 that had been digested with EcoRI and HindIII.

*Vectors for Tn7 insertion*

Plasmid pUC18T-mini-Tn7T-hph-Ptac (Gebhardt lab plasmid pMJG504) and plasmid pUC18T-mini-Tn7T-hph-Para (Gebhardt lab plasmid pMJG503) were created by amplifying the hygromycin resistance gene from plasmid pMJG111 (pUC18T-mini-Tn7T-hph) using primers MJG2690 and MJG2691. The resulting PCR product was ligated into XbaI-digested plasmids pJM101 (Ptac) and pJM100 (Para) using T4 DNA Ligase (NEB cat. no. M0202S) at room temperature for 15 minutes prior to heat shock transformation. The resulting clones were screened by colony PCR and Sanger sequencing to ensure correct orientation of the *hph* gene. Note that XbaI digestion of pJM100/101 removes the gentamicin resistance cassette harbored by these plasmids. Plasmid pUC18T-mini-Tn7T-hph-Ptac-VSV-G (Gebhardt lab plasmid pMJG520) was created by digesting plasmid pUC18T-mini-Tn7T-hph-Ptac with PstI and HindIII and inserting a PCR product (designed to allow for creation of C-terminal-VSV-G alleles using an in-frame NotI restriction site) amplified with primers MJG2717 and MJG2718. The Tn7-Ptac-Oxa23-VSVG plasmid (pMJG520 + Oxa23; Gebhardt lab plasmid number pMJG570) was created by digesting pUC18T-mini-Tn7T-hph-Ptac-VSV-G with PstI and NotI and subsequently inserting a PCR product containing the *oxa23* gene (ABUW_0563, construct includes 55 bp of DNA upstream of the annotated translation start codon and ends at the last amino acid in the open reading frame) using primers MJG2826 and MJG2793. Plasmid pUC18T-mini-Tn7T-hph-Ptol (Gebhardt lab plasmid pMJG608) was constructed from pMJG561-Ptol (pMJG587; described in the Tn7-lacZ plasmids section below) by digesting the parent plasmid (pMJG587) with PstI and XhoI and collecting the 6.1 kb fragment following agarose gel electrophoresis. Subsequently, a PCR product containing the multiple cloning site (MCS) from plasmid pJM100 was generated with primers MJG2925 and MJG2784 was inserted into the digested backbone via ITA as described above. Plasmid pUC18T-mini-Tn7T-hph-Ptet1 (Gebhardt lab plasmid pMJG607) was constructed from pMJG561-Ptet1 (pMJG585; described in the Tn7-lacZ plasmids section below) by digesting the parent plasmid (pMJG585) with PstI and XhoI and collecting the 4.8 kb fragment following agarose gel electrophoresis. Subsequently, a PCR product containing the multiple cloning site (MCS) from plasmid pJM100 was generated with primers MJG2926 and MJG2784 was inserted into the digested backbone via ITA as described above. Plasmid pUC18T-mini-Tn7T-hph-Ptet2 (Gebhardt lab plasmid pMJG617) was constructed from pMJG561-Ptet2 with PstI and XhoI and collecting the 4.9 kb fragment following agarose gel electrophoresis. Subsequently, a PCR product containing the multiple cloning site (MCS) from plasmid pJM100 was generated with primers MJG2927 and MJG2784 was inserted into the digested backbone via ITA as described above.

*Vectors for Tn7-lacZ fusions*

Plasmid pUC18T-mini-Tn7T-hph-lacZ (Gebhardt lab plasmid pMJG561) was created from pUC18T-mini-Tn7T-hph-Ptac (pMJG504). Briefly, the lacI^q^-Ptac regulatory region from plasmid pMJG504 was removed by digestion with NruI and the resulting 4.6 kb band was purified following agarose gel electrophoresis. The transcriptional-lacZ construct was amplified from plasmid pMini-CTX-lacZ in two pieces using PCR products generated with primers MJG2821 and MJG2789 (5′ portion of lacZ) and MJG2786 and MJG2787 (3′ portion of lacZ), which were inserted into the NruI-digested pMJG504 backbone by ITA. Plasmid pUC18T-mini-Tn7T-hph-lacZ-Ptet1 (Gebhardt lab plasmid pMJG585) was constructed by inserting a PCR product specifying TetR-PtetA-1x-tetO (generated with primers MJG2901 and MJG2902 using pYDE09 as template) into plasmid pMJG561 that had been digested with BamHI and PstI. Plasmid pUC18T-mini-Tn7T-hph-lacZ-Ptet2 (Gebhardt lab plasmid pMJG586) was constructed by inserting a PCR product specifying TetR-PtetA-2x-tetO (generated with primers MJG2901 and MJG2903 using pYDE09 as template) into plasmid pMJG561 that had been digested with BamHI and PstI. Plasmid pUC18T-mini-Tn7T-hph-lacZ-Ptol (Gebhardt lab plasmid pMJG587) was constructed by inserting a PCR product specifying XylS-Pm (generated with primers MJG2899 and MJG2900 using pTOL as template) into plasmid pMJG561 that had been digested with BamHI and PstI. Plasmid pUC18T-mini-Tn7T-hph-lacZ-Ptac (Gebhardt lab plasmid pMJG609) was constructed by inserting a PCR product specifying lacI^q^-Ptac (generated with primers MJG2937 and MJG2938 using pMJG504 as template) into plasmid pMJG561 that had been digested with BamHI and PstI. Plasmid pUC18T-mini-Tn7T-hph-lacZ-Para (Gebhardt lab plasmid pMJG646) was constructed by inserting a PCR product specifying araC-ParaBAD (generated with primers MJG3029 and MJG3030 using pMJG503 as template) into plasmid pMJG561 that had been digested with BamHI and PstI.

*CRISPRi plasmids*

The dCas9 delivery plasmid, pUC18T-mini-Tn7T-hph-dCas9 (Gebhardt lab plasmid pKEV39) was generated by subcloning the dCas9 cassette into plasmid pMJG111. Briefly, plasmid pYDE09 was digested with ApaI and SpeI to generate a 5.1 kb fragment containing the TetR-PtetA-2x-tetO-dCas9 cassette, which was gel purified and ligated into pMJG111 cut with the same enzymes. The sgRNA expression plasmid, pWH1266-Apra-sgRNA (Gebhardt lab plasmid pMJG440) was created by digesting plasmid pYDE07 with EcoRI and PstI, which removes the bulk of the bla-TEM gene. An apramycin resistance cassette, which was amplified from plasmid pMJG120 with primers MJG2343 and MJG2342, was subsequently introduced into the pYDE07 backbone via ITA. The sgRNA constructs were amplified using a common reverse primer, primer MJG2417, which incorporates the Cas9 handle and transcription terminator, and an sgRNA-specific forward primer, which was designed as specified by Geisigner and colleagues (2); see also Table S5. For the sgRNA targeting *oxa23*, the sgRNA cassette was amplified using primers MJG2414 and MJG2417 using pMJG440 as template. For the sgRNA targeting *hfq*, the sgRNA cassette was amplified using primers MJG2418 and MGG2417 using pMJG440 as template. For the sgRNA targeting *ABUW_0747*, the sgRNA cassette was amplified withing primers MJG2797 and MJG2417 using pMJG440 as template. The sgRNA cassettes were inserted into plasmid pWH1266-apra-sgRNA (pMJG440) that had been digested with SpeI and ApaI. Cloning and propagation of sgRNA constructs was performed at or below 30˚C in *E. coli* DH5α, as growth at higher temperatures led to the isolation of mutations to the promoter region found in plasmid pMJG440; the J23119 promoter driving sgRNA expression is highly active in *E. coli*, and we hypothesize that growth at higher temperature is toxic to DH5α.

*Plasmid stability testing*

Colonies (n = 3) of *A. baumannii* AB5075 harboring pMApra-Ptac or pMApra-Ptac-mCherry were grown overnight in 5 mL LB with apramycin. The resulting cultures were back-diluted 1:200 in fresh growth LB media in the presence or absence of apramycin and the presence or absence of 2 mM IPTG and grown at 37˚C for 24 hours in a SpectraMax 190 96-well plate reader with shaking every 20 minutes. Following the 24-hour growth period, cultures were serially diluted in sterile PBS and plated onto LB agar plates and LB agar plates containing apramycin. Plasmid stability was determined as the fraction of apramycin-resistant colonies divided by the total colonies. Assay was repeated twice with biological triplicate cultures. Results from a single representative experiment are shown.

**Supplemental Figures**

**Figure S1**

**Figure S1.** Multiple cloning sites in pMApra plasmids. Unique restriction sites are shown for each of the pMApra derivatives described in the main text. Letters in bold font indicate transcription start sites (TSS) for the regulatory systems. The TSS for those plasmids that do not have an indicated TSS are positioned 5′ to the MCS.

**Figure S2**

**Figure S2.** Growth curve of cells harboring pMApra derivatives with and without induction. Figure accompanies Figure 2 in the main text. Growth curves performed in the presence and absence of inducers for IPTG-inducible vectors (A), tetracycline-inducible vectors (B), and toluic acid-inducible vectors (C) and arabinose-inducible vectors (D). Growth curves were performed with biological triplicate cultures and were repeated three times with independent transformations for each. Solid lines indicate growth in LB or LB containing vehicle control: DMSO for aTc experiments (Panel B) Dimethyl formamide (DMF) for toluate experiments (Panel C). Dashed lines indicate cultures containing corresponding inducer.

**Figure S3**

**Figure S3. Plasmid stability testing for pMApra-Ptac.** Cells harboring pMApra-Ptac or pMApra-Ptac-mCherry were grown in the presence or absence of apramycin and in the presence or absence of 2 mM IPTG. Overnight cultures (in LB + apramycin) were refreshed into LB, LB plus apramycin, LB plus IPTG, or LB plus apramycin and 2 mM IPTG and grown for 24 hr in a 96-well dish at 37˚C. Following the outgrowth, the cultures were serially diluted and plated on LB agar plates or LB agar plates with apramycin. CFU were enumerated on each plate and plasmid stability was determined by dividing the number of pMApra-containing colonies (i.e., # CFU on LB + apramycin plates) by the total colonies (i.e., # CFU on LB plates). Data is plotted as the mean plasmid stability across biological triplicate cultures; stability experiments were repeated twice with freshly transformed AB5075. Statistics determined between stability in LB compared to LB + IPTG by two-tailed t-test; *, p < 0.05; ns, not significant.

**Figure S4**

**Figure S4.** Multiple cloning sites in pUC18T-miniTn7T-hph plasmids. Unique restriction sites are shown for the Tn7-delivery plasmids described in the main text. Letters in bold font indicate transcription start sites (TSS) for the regulatory systems. The TSS for those plasmids that do not have an indicated TSS are positioned 5′ to the MCS.

**Figure S5**.

**Figure S5**: CRISPRi plasmids. (A) Plasmid map for pUC18T-mini-Tn7T-hph-dCas9. Dark blue arrows indicate Flp recombinase target sites; red octagons indicate transcription terminators; light blue circles represent the left and right ends of the Tn7 cassette; *hph*, hygromycin resistance gene; *bla*, beta-lactamase resistance gene; *oriV*, plasmid origin of replication; *oriT*, origin of transfer. (B) Plasmid map for sgRNA expression plasmid pWH1266-Apra-sgRNA. The apramycin resistance gene (*aac(3)IV*) was inserted into the 5′ end of the *bla* beta-lactamase of plasmid pYDE07 (2). (C) sgRNA expression cassette (boxed region in panel B). (D) Western blot analysis of WT AB5075 and AB5075 attTn7::*dCas9.* Cells were grown until mid-exponential phase (OD_600_ ≈ 0.5), at which time anhydrotetracycline was added at 200 ng/mL for three additional hours to induce expression of dCas9. Asterisk indicates the expected migration of the dCas9 protein (molecular weight ≈ 158 kDa); faster migrating bands are non-specific.

**Supplementary Tables**

**Table S1. Bacterial strains**

| **Strain** | **Description** | **Reference** |
| --- | --- | --- |
| *Escherichia coli* | |  |
| DH5α F′-lacI^q^ | Cloning strain | Lab Stock |
| LW264 | Donor strain for mobilizing plasmids into *A. baumannii* | (3) |
| SM10 pir^+^ pTNS3 | Tn7 mating helper plasmid | Lab Stock |
| Top10 pRK2013 | Tn7 mating helper plasmid | Lab Stock |
| *Acinetobacter baumannii* | |  |
| AB5075-UW | Multidrug resistant clinical isolate | (4) |

**Table S2. Plasmids used in this study**

| **Plasmid** | **Description** | | | | **Resistance** | **Source** |
| --- | --- | --- | --- | --- | --- | --- |
| *Source plasmids* | | | | | | |
| pJM100 | pUC18T-mini-Tn7T-gm-araC-ParaBAD | | | | Gent | (5) |
| pJM101 | pUC18T-mini-Tn7T-gm-lacI^q^-Ptac | | | | Gent | (5) |
| pMJG111 | pUC18T-mini-Tn7T-hph | | | | Hyg | (6) |
| pYDE07 | pWH1266-pgRNA; sgRNA expression vector | | | | Carb | (2) |
| pYDE09 | pUC18T-mini-Tn7T-Gm-dCas9; source for dCas9 | | | | Gent | (2) |
| pMini-CTX-lacZ | Source for transcriptional *lacZ* fusions | | | | Tet | (7) |
| pTOL | pMMB66EH derivative with XylS-Pm | | | | Carb | Arne Reitsch |
| pKH6 | pBBR1-Gm-AraC-P_BAD_; source for AraC-P_BAD_ | | | | Gent | (8) |
| pXDC18.mCherry | pMMB207c-gent-mCherry; source for mCherry | | | | Gent | (9) |
|  |  | | | |  |  |
| *Empty vectors for multi-copy plasmids* | | | | | | |
| pMJG120 | pMApra-Ptac | LacI^q^-Ptac | | | Apra | (3) |
| pMJG584 | pMApra-Plac-SO | LacI^q^-Plac-SO | | | Apra | This study |
| pMJG588 | pMApra-Ptol | XylS-Pm | | | Apra | This study |
| pMJG598 | pMApra-Ptet1 | TetR-PtetA-1x-tetO | | | Apra | This study |
| pMJG599 | pMApra-Ptet2 | TetR-PtetA-2x-tetO | | | Apra | This study |
| pMJG464 | pMApra-Para | araC-ParaBAD | | | Apra | This study |
|  |  | | | |  |  |
| *mCherry Expression vectors* | | | | | | |
| pMJG116 | pMJG120 + mCherry | LacI^q^-Ptac | | | Apra | This study |
| pMJG596 | pMJG584 + mCherry | LacI^q^-Plac-SO | | | Apra | This study |
| pMJG602 | pMJG588 + mCherry | XylS-Pm | | | Apra | This study |
| pMJG603 | pMJG598 + mCherry | TetR-PtetA-1x-tetO | | | Apra | This study |
| pMJG604 | pMJG599 + mCherry | TetR-PtetA-2x-tetO | | | Apra | This study |
| pMJG484 | pMJG464 + mCherry | araC-ParaBAD | | | Apra | This study |
|  |  | | | |  |  |
| *Empty vectors for Tn7 insertion with regulatory control* | | | | | | |
| pMJG504 | pUC18T-mini-Tn7T-hph-Ptac | | LacI^q^-Ptac | | Hyg | This study |
| pMJG520 | pUC18T-mini-Tn7T-hph-Ptac-VSV-G | | | LacI^q^-Plac | Hyg | This study |
| pMJG608 | pUC18T-mini-Tn7T-hph-Ptol | | XylS-Pm | | Hyg | This study |
| pMJG607 | pUC18T-mini-Tn7T-hph-Ptet1 | | TetR-PtetA-1x-tetO | | Hyg | This study |
| pMJG617 | pUC18T-mini-Tn7T-hph-Ptet2 | | TetR-PtetA-2x-tetO | | Hyg | This study |
| pMJG503 | pUC18T-mini-Tn7T-hph-Para | | araC-ParaBAD | | Hyg | This study |
|  |  | | | |  |  |
| *dCas9 Expression plasmid* | | | | | | |
| pKEV39 | pMJG111 + dCas9 cassette | | | | Hyg | This study |
|  |  | | | |  |  |
| *Oxa23-VSV-G Expression plasmid* | | | | | | |
| pMJG570 | pMJG520 + Oxa23 | | | | Hyg | This study |
|  |  | | | |  |  |
| *Transcriptional lacZ fusion plasmids* | | | | | | |
| pMJG561 | pUC18T-mini-Tn7T-hph-lacZ | | | | Hyg | This study |
| pMJG585 | pMJG561 + Ptet1 (TetR-PtetA-1x-tetO) | | | | Hyg | This study |
| pMJG586 | pMJG561 + Ptet2 (TetR-PtetA-2x-tetO) | | | | Hyg | This study |
| pMJG587 | pMJG561 + Ptol (XylS-Pm) | | | | Hyg | This study |
| pMJG609 | pMJG561 + Ptac (lacI^q^-Ptac) | | | | Hyg | This study |
| pMJG646 | pMJG561 + Para (araC-ParaBAD) | | | | Hyg | This study |
|  |  | | | |  |  |
| *CRISPRi sgRNA plasmids* | | | | | | |
| pMJG440 | pYDE07 converted to apramycin resistance | | | | Apra | This study |
| pKEV40 | pMJG440 + oxa23-sgRNA | | | | Apra | This Study |
| pKEV41 | pMJG440 + hfq-sgRNA | | | | Apra | This study |
| pMJG557 | pMJG440 + ABUW_0747-sgRNA | | | | Apra | This study |

**Table S3. Oligonucleotide Primers**

| **Oligo** | **Purpose** | **Sequence (5′ – 3′)^a^** |
| --- | --- | --- |
| *Oligos for pMMB-Apra plasmids* | | |
| MJG2442 | Fwd for AraC-ParaBAD | GCAATTCGCGCTAACTTACATTAATCTCGAGGTCGACGGTATCGATGC |
| MJG2443 | Rev for AraC-ParaBAD | TTCCCTACTCTCGCATGGGGAGACCTATAAAAAATCCCGAAACCGTTATGCAGGCTCTAACTATTACCTGCGAACTGTTTCGGGATTCAGCCACTAGTAAGCTTCTGCAGGA |
| MJG2874 | Fwd for lacIq | GTTACTGGTTTCACATTCACCACCCTG |
| MJG2875 | Rev for lacIq | gcagggaaCGAGAAAAACATTATCCAGAACGGGAGTG |
| MJG2876 | Fwd for SO-Plac | ggataatgtttttctcgTTCCCTGCTGCCTGACGTG |
| MJG2878 | Rev for SO-Plac | GGGATTCAGCCACTAGTAAGCTTCTGCAGGAATTCGACG |
| MJG2897 | Fwd for xylS-Pm | gcaattcgcgctaacttacattaatCGTTCGTAATCAAGCCACTTCCTTT |
| MJG2898 | Rev for xylS-Pm | gtaagcttctgcaggaattcgacgTggatccAGCCTAAGGGGTAGGCCTTTC |
| MJG2917 | Fwd for tetR-PtetA | caattcgcgctaacttacattaattTTAAGACCCACTTTCACATTTAAGTTGT |
| MJG2918 | Rev for tetR-PtetA-1x-tetO | agcttctgcaggaattcgacgtctttaaaGGTAAAATAACTCTATCAACGATAGAGTGTC |
| MJG2919 | Rev for tetR-PtetA-2x-tetO | tctgcaggaattcgacgtctctagaTTTTCTCTATCACTGATAGGGAGTGG |
| MJG2848 | Fwd for mCherry (lacI^q^-SO-Plac) | ataacaattataattctagaTGAATTCTTTAAGAAGGAGATATACATATGGTTTCCAAGG |
| MJG2849 | Rev for mCherry (lacI^q^-SO-Plac) | tcgggattcagccactagtaagcttTTATTTGTACAGCTCATCCATGCCACC |
| MJG2904 | Fwd for mCherry (XylS-Pm) | ctaccccttaggctggatccTGAATTCTTTAAGAAGGAGATATACATATGGTTTCCAAGG |
| MJG2923 | Fwd for mCherry (PtetA-1x-tetO) | ttgatagagttattttaccttGAATTCTTTAAGAAGGAGATATACATATGGTTTCCAAGG |
| MJG2924 | Fwd for mcherry (PtetA-2x-tetO) | gagaaaatctagagacgtcGAATTCTTTAAGAAGGAGATATACATATGGTTTCCAAGG |
| MJG2690 | Fwd for *hph* gene | atatctagagaataggaacttcggaataggaacttcAACACCAGCGACAGCCGAGC |
| MJG2691 | Rev for *hph* gene | tattctagaaagtataggaacttcAGCTAGAGGGGGCGTCAGGC |
| MJG2717 | Fwd for CTD-VSVG (Tn7) | gaaacagactagtgctctgcagGCGGCCGCTTACACAGATATTGAAATGAACCGTTTAGG |
| MJG2718 | Rev for CTD-VSVG (Tn7) | gcgaggtaccgggcccaagcttTCATTATTACTTACCTAAACGGTTCATTTCAATATCTG |
| MJG2826 | Fwd for oxa23-VSVG (Tn7) | caggaaacagactagtgctctgcagGAGTTATCTATTTTTGTCGTGTACAGAGTTAT |
| MJG2793 | Rev for oxa23-VSG (Tn7) | aatatctgtgtaagcggccgcAATAATATTCAGCTGTTTTAATGATTTCATCAATAATTC |
| MJG2925 | Fwd for XylS-Pm MCS (Tn7) | aggcctaccccttaggctggatccaCTGCAGGAATTCCTCGAGAAGCTTG |
| MJG2926 | Fwd for PtetA-1x-tetO MCS (Tn7) | ttgatagagttattttaccactcccCTGCAGGAATTCCTCGAGAAGCTTG |
| MJG2927 | Fwd for PtetA-2x-tetO MCS (Tn7) | gatagagaaaagaattcaaaagatcCTGCAGGAATTCCTCGAGAAGCTTG |
| MJG2821 | Fwd for 5′-lacZ | ttcccggggatccgcatgcctgcagTAACTAACTAGCGATCCCGACTCACTAT |
| MJG2789 | Rev for 5′-lacZ | CAGCAACGAGACGTCACGGAAAATG |
| MJG2786 | Fwd for 3′-lacZ | CATTTTCCGTGACGTCTCGTTGCTG |
| MJG2787 | Rev for 3′-lacZ | CACTTATCTGGTTGGCCTGCAAGG |
| MJG2901 | Fwd for TetR-PtetA-lacZ | catgagctcgaattcccggggatccATGCATGAGCTCACTAGTTTAAGAC |
| MJG2902 | Rev for PtetA-1x-tetO-lacZ | cgggatcgctagttagttactgcagGGGAGTGGTAAAATAACTCTATCAACG |
| MJG2903 | Rev for PtetA-2x-tetO-lacZ | cgggatcgctagttagttactgcagGATCTTTTGAATTCTTTTCTCTATCACTGA |
| MJG2899 | Fwd for XylS-Pm-lacZ | catgagctcgaattcccggggatccCGTTCGTAATCAAGCCACTTCCTTT |
| MJG2900 | Rev for XylS-Pm-lacZ | cgggatcgctagttagttactgcagTggatccAGCCTAAGGGGTAGGCCTTTC |
| MJG2937 | Fwd for lacI^q^-Ptac-lacZ | catgagctcgaattcccggggatccCTTGCAATTCGCGCTAACTTACATTAATTGCG |
| MJG2938 | Rev for lacI^q^-Ptac-lacZ | cgggatcgctagttagttactgcagAGCACTAGTCTGTTTCCTGTGTGAAATTGT |
| MJG3029 | Fwd for AraC-ParaBAD-lacZ | catgagctcgaattcccggggatccGAATCCCCAAATTATGACAACTTGACGGC |
| MJG3030 | Rev for AraC-ParaBAD-lacZ | cgggatcgctagttagttactgcagAGCACTAGTCTAGCCCAAAAAAACGGG |
| MJG2342 | Fwd for Apra-R (for pYDE07) | ataagctgtcaaacatgagaattcATCAAGGCCCGATCCTTGGAG |
| MJG2343 | Rev for Apra-R (for pYDE07) | gcaacgttgttgccattgctgcagTCATGAGCTCAGCCAATCGACTGG |
| MJG2417 | Rev for sgRNA | GAGATGAGTTTTTGTTCGGGCCCAA |
| MJG2414* | Fwd for oxa23 sgRNA | ctcagtcctaggtataatactagtCTGTTTGAATAACCAGCACACCTGGTTTTAGAGCTAGAAATAGCAAG |
| MJG2418* | Fwd for hfq sgRNA | ctcagtcctaggtataatactagtTAGAAACTGGGATGCGTTCTTTAGTTTTAGAGCTAGAAATAGCAAG |
| MJG2797* | ABUW_0747 sgRNA fwd | gctcagtcctaggtataatactagtTTTGACCCCTCCAACTCTTGGGTTGTTTTAGAGCTAGAAATAGCAAG |

**Notes:** **a**, lower case font indicates homology regions included in primers to facilitate isothermal assembly.

*, for sgRNA fwd primers, the sgRNA sequence is underlined.

**Table S4. Synthetic DNA constructs (gBlocks)**

| **gBlock** | **Purpose** | **Sequence (5′ – 3′)** |
| --- | --- | --- |
| Twin_operator_v1 | Template for single-operator lac promoter | CGTTACTGGTTTCACATTCACCACCCTGAATTGACTCTCTTCCGGGCGCTATCATGCCATACCGCGAAAGGTTTTGCACCATTCGATGGTGTCAACGTAAATGCCGCTTCGCCTTCGCGCGCGAATTGCAAGCTGATCCGGGCTTATCGACTGCACGGTGCACCAATGCTTCTGGAGTCAGGCAGCCATCGGAAGCTGTGGTATGGCTGTGCAGGTCGTAAATCACTGCATAATTCGTGTCGCTCAAGGCGCACTCCCGTTCTGGATAATGTTTTTCTCGAGGGTAAATGTGAGCACTCACAATTTATTCCCTGCTGCCTGACGTGAGCTCATTTATTCTGAAATGAGCTCTTGAAATTGTGAGCGGATAACAATTATAATTCTAGAGACGTCGAATTCCTGCAGA |

**Table S5. sgRNAs for CRISPRi**

| **Target Gene** | **sgRNA target sequence (5′ - 3′)** | **Target start** | **Target end** | **PAM** | **Seed #** | **Strand targeted** |
| --- | --- | --- | --- | --- | --- | --- |
| *oxa23* | CTGTTTGAATAACCAGCACACCTG | 563146 | 563169 | CCT | 1 | NT |
| *hfq* | TAGAAACTGGGATGCGTTCTTTA | 3660292 | 3660314 | CCG | 1 | NT |
| *ABUW_0747* | TTTGACCCCTCCAACTCTTGGGTT | 747961 | 747984 | CCC | 1 | NT |

Notes: Target gene = gene targeted by sgRNA.

sgRNA targeting sequence (5′-3′) = 5'-3' sequence of the targeting region cloned within the sgRNA module in pMJG440

Target start = position in chromosome NZ_CP008706 of leftmost end of sgRNA targeting region

Target end = position in chromosome NZ_CP008706 of rightmost end of sgRNA targeting region

PAM = Protospacer Adjacent Motif (PAM) sequence as read 5'-3' in top strand of chromosome NZ_CP008706.

Seed # = Number of unique occurrences in AB5075-UW genome (chromosome + plasmids) of the sgRNA 12-bp seed region next to a PAM sequence (NGG)

Strand targeted = strand (NT, non-template) targeted by sgRNA

**Supplemental References**

1. Gibson DG, Young L, Chuang RY, Venter JC, Hutchison CA, 3rd, Smith HO. 2009. Enzymatic assembly of DNA molecules up to several hundred kilobases. Nat Methods 6:343-5.

2. Bai J, Dai Y, Farinha A, Tang AY, Syal S, Vargas-Cuebas G, van Opijnen T, Isberg RR, Geisinger E. 2021. Essential Gene Analysis in *Acinetobacter baumannii* by High-Density Transposon Mutagenesis and CRISPR Interference. J Bacteriol 203:e0056520.

3. Gebhardt MJ, Gallagher LA, Jacobson RK, Usacheva EA, Peterson LR, Zurawski DV, Shuman HA. 2015. Joint Transcriptional Control of Virulence and Resistance to Antibiotic and Environmental Stress in *Acinetobacter* *baumannii*. mBio 6:e01660-15.

4. Jacobs AC, Thompson MG, Black CC, Kessler JL, Clark LP, McQueary CN, Gancz HY, Corey BW, Moon JK, Si Y, Owen MT, Hallock JD, Kwak YI, Summers A, Li CZ, Rasko DA, Penwell WF, Honnold CL, Wise MC, Waterman PE, Lesho EP, Stewart RL, Actis LA, Palys TJ, Craft DW, Zurawski DV. 2014. AB5075, a Highly Virulent Isolate of *Acinetobacter* *baumannii*, as a Model Strain for the Evaluation of Pathogenesis and Antimicrobial Treatments. MBio 5:e01076-14.

5. Meisner J, Goldberg JB. 2016. The *Escherichia coli rhaSR-PrhaBAD* Inducible Promoter System Allows Tightly Controlled Gene Expression over a Wide Range in *Pseudomonas aeruginosa*. Appl Environ Microbiol 82:6715-6727.

6. Gebhardt MJ, Czyz DM, Singh S, Zurawski DV, Becker L, Shuman HA. 2020. GigC, a LysR Family Transcription Regulator, Is Required for Cysteine Metabolism and Virulence in *Acinetobacter baumannii*. Infect Immun 89:e00180-20.

7. Becher A, Schweizer HP. 2000. Integration-proficient *Pseudomonas aeruginosa* vectors for isolation of single-copy chromosomal *lacZ* and *lux* gene fusions. Biotechniques 29:948-952.

8. Han K, Tjaden B, Lory S. 2016. GRIL-seq provides a method for identifying direct targets of bacterial small regulatory RNA by in vivo proximity ligation. Nat Microbiol 2:16239.

9. Walker AC, Bhargava R, Vaziriyan-Sani AS, Pourciau C, Donahue ET, Dove AS, Gebhardt MJ, Ellward GL, Romeo T, Czyż DM. 2021. Colonization of the *Caenorhabditis elegans* gut with human enteric bacterial pathogens leads to proteostasis disruption that is rescued by butyrate. PLoS Pathog 17:e1009510.
